# Case Report: Effects of multiple myeloma therapy on essential thrombocythemia and vice versa: a case of synchronous dual hematological malignancy

**DOI:** 10.3389/fonc.2023.1213942

**Published:** 2023-06-08

**Authors:** Nupur Krishnan, Russell Price, Rouslan Kotchetkov

**Affiliations:** ^1^ Department of Medical Sciences, University of Western Ontario, London, ON, Canada; ^2^ Simcoe Muskoka Regional Cancer Program, Royal Victoria Regional Health Centre, Barrie, ON, Canada

**Keywords:** essential thrombocythemia, multiple myeloma, synchronous dual hematological malignancy, calreticulin (CALR), monoclonal protein

## Abstract

**Background:**

Dual hematological malignancies, asynchronous or synchronous, are underrecognized entities and are usually suspected when clinical, hematological, or biochemical features cannot be explained by the primary malignancy alone. We present a case of synchronous dual hematological malignancies (SDHMs), where the patient was diagnosed with symptomatic multiple myeloma (MM) and essential thrombocythemia (ET), when excessive thrombocytosis occurred following initiation of MPV (melphalan–prednisone–bortezomib) antimyeloma therapy.

**Case description:**

An 86-year-old woman presented to the emergency in May 2016 with confusion, hypercalcemia, and acute kidney injury. She was diagnosed with free light chain (FLC) lambda and Immunoglobulin G (IgG) lambda MM and started MPV (standard of care at that time) treatment with darbopoietin support. At diagnosis, she had normal platelet count since the ET was likely masked by bone marrow suppression due to active MM. After she reached stringent complete remission with no MP detected on serum protein electrophoresis or immunofixation, we noticed that her platelet counts increased to 1,518,000 × 10^9^/L. She was tested positive for mutation in exon 9 of calreticulin (CALR). We concluded that she had concomitant CALR-positive ET. After bone marrow recovery from MM, the ET became clinically apparent. We started hydroxyurea for ET. Treatment for MM with MPV did not affect the course of ET. Presence of concomitant ET did not decrease the efficacy of sequential antimyeloma therapies in our elderly and frail patient.

**Conclusion:**

The possible mechanism underlying the occurrence of SDHMs is unclear but is likely due to stem cell differentiation defects. SDHMs can be challenging to treat and warrant several considerations. In the absence of clear guidelines on how to manage SDHMs, management decisions depend on several factors including disease aggressiveness, age, frailty, and comorbidities.

## 1 Introduction

Dual malignancies occurring in the same patient are reported in the literature (1); however, dual hematological malignancies (DHMs) are recognized less frequently (2–5) and are likely underreported (6). DHMs can been classified as either synchronous (SDHMs), when occurring within 6 months of diagnosis of the first malignancy, or asynchronous when occurring later (1). Using a more restrictive cutoff of 1 month, we had earlier reported a 1.5% incidence of SDHMs in patients referred to our cancer center (6). The detection of DHMs may be an incidental finding during routine bloodwork or during investigation of discrepant clinical and laboratory findings (6). The observed underreporting, and potential under detection, of SDHMs may be because of masking from the primary malignancy (6). DHMs involving essential thrombocythemia (ET) and multiple myeloma (MM) are quite uncommon, and most cases report MM developing years after ET diagnosis. The occurrence of these two malignancies synchronously is extremely rare. We report a case of concurrent MM and ET in a frail elderly patient. We review the literature, discuss possible mechanisms, and present potential challenges in the management of such patients.

## 2 Case presentation

### 2.1 Patient information/clinical findings

**Figure 1:**
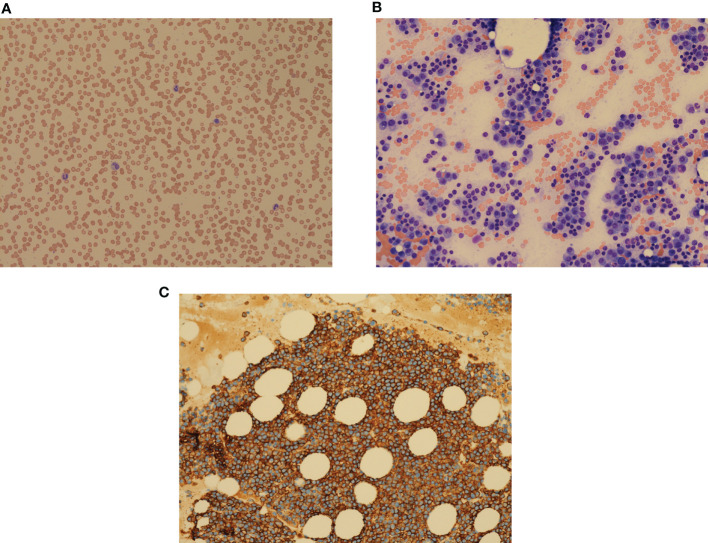
**(A)** Peripheral blood film showing multiple rouleaux, morphologically normal WBC, and platelets. May–Giemsa, ×100 objective. **(B)** Bone marrow aspirate with numerous plasma cells, comprising over 90% of bone marrow cells, reduced erythropoiesis, granulopoiesis, and thrombopoiesis. Plasma cells contain Dutcher bodies and Russell bodies. H&E, ×200 objective. **(C)** Strong expression of CD138 on plasma cell surface. Clot section, H&E, ×200 objective.

An 86-year-old woman presented to the emergency room in May 2016 with confusion, hypercalcemia, and acute kidney injury. Her past medical history included hypertension, hyperlipidemia, and osteoporosis. She was brought to the clinic on a stretcher with an Eastern Cooperative Oncology Group (ECOG) status of 4. On examination, she was disoriented and had tenderness along her left rib cage. Investigations showed hemoglobin (Hb) 82 g/L [normal 115–160], macrocytosis (Mean corpuscular volume (MCV) 134, [normal 80–95fl), rouleaux, White blood cell (WBC) 4.2 × 10^9^/L [normal 4.0–11.0] with normal differential, and platelets 226 × 10^9^/L [normal 150–400] (**Figure 1A**). Chemistry showed total protein 99 g/L [normal 60–80], albumin 26 g/L [normal 40.2–47.6], calcium 3.15 mmol/L [normal 2.1–2.6], and creatinine clearance 17 ml/min [normal 100–130]. Total IgG was elevated (45.9 g/L, [normal 5.28–21.90]) with reciprocally decreased IgA and IgM. Monoclonal protein (MP) was 41.8 g/L. Free light chain (FLC) lambda was elevated (9,050 mg/L, [normal 5.6–26.3]). A skeletal survey showed osteopenia and a T12 vertebral compression fracture. Bone marrow (BM) examination showed infiltration by plasma cells (PCs) comprising up to 90% of nucleated cells including occasional binuclear and atypical PCs (**Figure 1B**). The PCs were kappa-restricted and had strong cytoplasmic CD138+ expression (**Figure 1C**). Erythroid and megakaryocytic maturation was reduced but normal morphologically.

### 2.2 Diagnostic assessment/therapeutic intervention

At that stage, the patient was presenting with the classical picture of FLC lambda and IgG lambda MM, standard risk. Fluorescence *in situ* hybridization was positive for monosomy 13/13q deletion and no high-risk abnormalities, like del(17p) or t (4; 14) and/or t (14; 16), were found. The Revised International Staging System was II. Since her platelet count was normal, no workup for myeloproliferative neoplasms was done.

She started melphalan–prednisone–bortezomib (MPB) treatment with darbepoetin support and zoledronic acid in June 2016. By October 2016, her Hb improved to 132 g/L and FLC decreased to 9.7 mg/L. She reached stringent complete remission (CR) with no MP detected on serum protein electrophoresis or immunofixation (IFE). We noticed that her platelet counts increased to 514 × 10^9^/L in July, 810 × 10^9^/L in September, and 1,518 × 10^9^/L in October. Presence of giant platelets in peripheral blood was also reported. The mean platelet volume (MPV) was elevated: 11.4 fl (upper limit of norm 11.0). These findings suggested association of thrombocytosis with increased interleukin-6 production by PCs.

Due to the appearance of unexpected thrombocytosis, we first ruled out infections and iron deficiency. The platelet count was unusually high for secondary causes for thrombocytosis; thus, we initiated workup for myeloproliferative neoplasms. DNA was extracted from the peripheral blood and analyzed by PCR for JAK2 and calreticulin (CALR) mutations. JAK2V617F mutation was negative, but she was found to have a mutation in exon 9 of CALR. We concluded that she had concomitant CALR-positive ET. Based on an International Prognostic Score for Essential Thrombocytopenia score of 4, she had high-risk ET. Due to classical ET presentation and the patient’s preference, we omitted repeated bone marrow examination. Aspirin and hydroxyurea (HU) were added to the MPV, stabilizing her platelet counts (300 × 10^9^/L). As this second diagnosis occurred within 5 months of diagnosis of the first malignancy, we updated her diagnosis to a synchronous dual hematological malignancy (SDHM): MM and ET, initially masked by marrow infiltration by PC.

**Figure 2:**
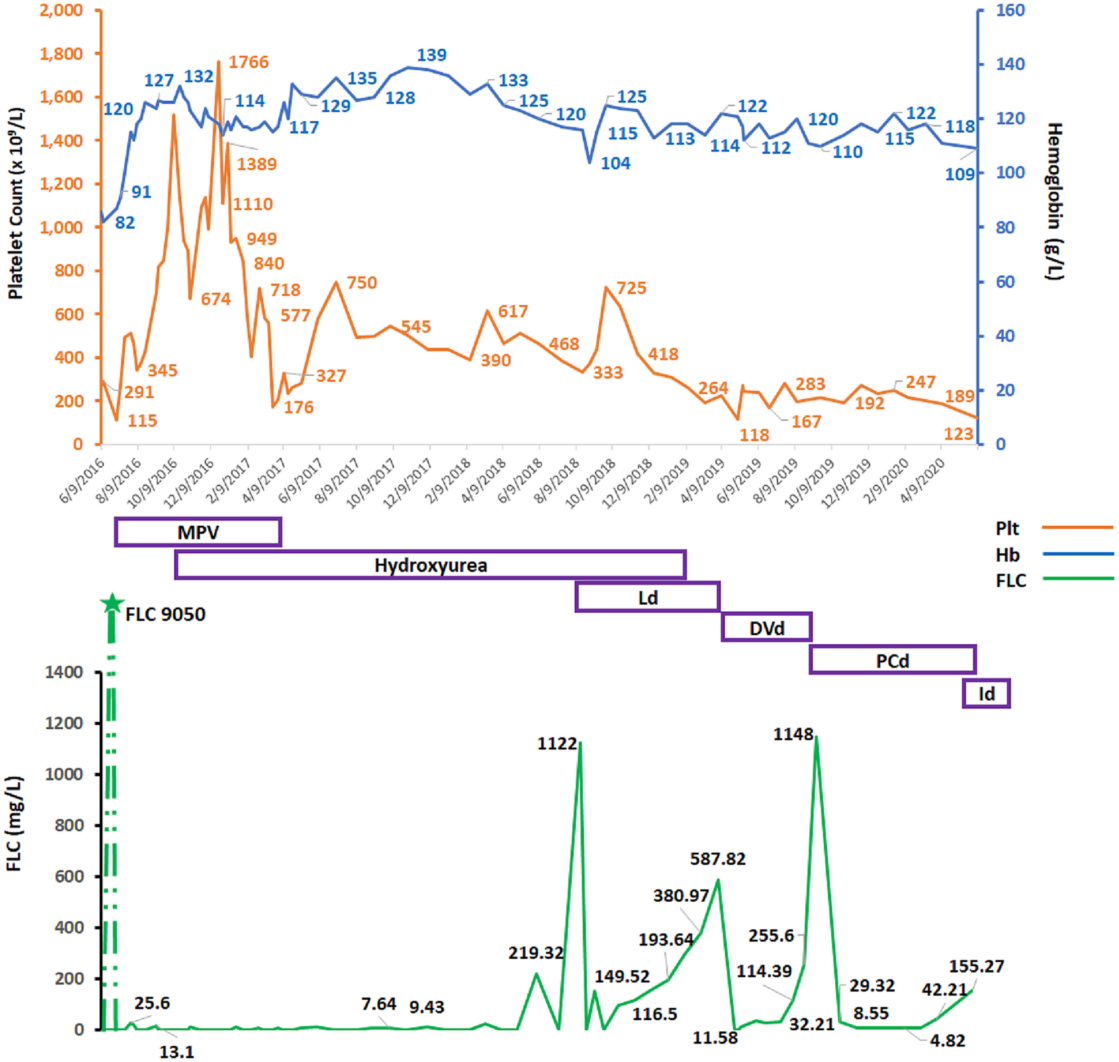
Graphical changes over time in patient’s, hemoglobin, platelet counts, and affected free light chain (FLC) over the course of disease and antimyeloma therapy. Hb, hemoglobin (g/L); Plt, platelet count (×10^9^/L), FLC, serum free light chain (mg/L); MPV, melphalan–prednisone–velcade (bortezomib); Ld, lenalidomide and dexamethasone; DVd, daratumumab–velcade (bortezomib)–dexamethasone; PCd, pomalidomide–cyclophosphamide–dexamethasone; Id, ixazomib–dexamethasone.

She finished the MPV in April 2017. By August 2018, her platelets were 388 × 10^9^/L; she relapsed, with FLC climbing to 1,130 mg/L, MP 23.9 g/L, and Hb dropping to 104 g/L. HU was held, and she was started on second-line lenalidomide–dexamethasone (Ld) treatment for MM. Upon achieving a second CR, her platelet count increased to 500 × 10^9^/L and HU was restarted. In March 2019, her MM progressed again with FLC increasing over 600 mg/L, and MP 17.0 g/L. She developed new anemia (Hb 114 g/L) and thrombocytopenia (platelets 118 × 10^9^/L). Once again, HU was held. She was started on third-line antimyeloma therapy with DVd (daratumumab–bortezomib–dexamethasone) in April 2019. FLC decreased to 11.5 mg/L, MP was down to 0.2 g/L, Hb increased to 120 g/L, and platelets increased (580 × 10^9^/L). She attained third remission, and we restarted HU. After the seventh cycle with DVd, however, her FLC increased again to 255 mg/L, MP was 6.9 g/L, and Hb decreased to 110 g/L. HU was put on hold, and platelets remained low-normal (154 × 10^9^/L). In October 2019, she started fourth-line therapy with PCd (pomalidomide–cyclophosphamide–dexamethasone), and she reached CR with no measurable MP and normalization of FLC kappa (6.6 mg/L) in December 2019. Platelet count was 247 × 10^9^/L. She was in remission until June 2020 when she progressed to PC leukemia and passed away. FLC and platelet count dynamics over the course of disease and treatment is shown in **Figure 2**.

## 3 Discussion

SDHMs are suspected if certain clinical, hematological, or biochemical features cannot be explained by the primary malignancy alone. These cases can be challenging to diagnose and treat and warrant additional attention and investigation. **Table 1** summarizes case reports of synchronous ET and MM (7–12). The median patient age was 59 years, with equal gender distribution (five men and three women). The median platelet count was 835 × 10^9^/L. Plasma cells in bone marrow ranged from 11.5% to 90%, as well as one patient who had only 6% PCs but presented with a plasmacytoma. Molecular markers were only reported for three cases and were all JAK2 positive. ET and MM were diagnosed concomitantly in all except one, in which ET diagnosis preceded that of MM by 1 month.

**Table 1:** Synchronous dual hematological malignancy: multiple myeloma (MM) and thrombocytosis.

Primary malignancy	Second malignancy	Time of 2nd malignancy	Age/Sex	Platelet count (×10^9^/L)	Bone marrow examination	MP	Molecular	Reference
MM	ET*	Concomitant	63/M	800	60% PC + Mega-K	NR	NR	Zimelman, 1973 (7)
MM	ET*	Concomitant	47/M	871	90% PC	NR	NR	Zimelman, 1973 (7)
PM	ET*	Concomitant	56/M	726	6% PC	IgG	NR	Zimelman, 1973 (7)
MM	ET	Concomitant	76/M	931	70% PC, Mega-K	IgD	NR	Nestler, 1981 (8)
ET	MM	Concomitant	83/F	910	Mega-K + 36% PC	FLC lambda	NR	Ghosh, 1984 (9)
ET	MM	Concomitant	67/F	113	11.5% PC	IgG lambda	JAK2+	Kuroda, 2008 (10)
MM	ET	Concomitant	51/M	9,000	Mega-K + 20% PC	NR	JAK2+	Holtan, 2011 (11)
ET	MM	1 Month	32/F	594	1st: hypocellular 2nd: Normal PET: lytic lesion	IgG lambda	JAK2+	Naeem, 2019 (12)

ET, essential thrombocythemia; PM, plasmacytoma; MM, multiple myeloma; MP, monoclonal protein; NR, not reported; Mega-K, megakaryocyte proliferation; PC, plasma cell; FLC, free light chain.

*Clinical diagnosis, no JAK status available at that time.

There are more reports of asynchronous MM and ET, as summarized in **Table 2** (13–24). The median patient age was 67 years, with nine men and five women. The median platelet count was 1,065 × 10^9^/L. Bone marrow PCs ranged from 24% to 100%. Molecular markers were only reported for five of the cases: three were JAK2 positive, while two were JAK2 negative. In all cases, ET preceded MM with a median of 4.5 years between diagnoses.

**Table 2:** Asynchronous dual hematological malignancy: MM and essential thrombocythemia.

Primary malignancy	Second malignancy	Time of 2nd malignancy	Age/Sex	Platelet count (×10^9^/L)	Bone marrow examination	MP	Molecular	Reference
ET	MM	6 Months	78/M	1,420	Mega-K and PC	IgA kappa	NR	Selroos, 1984 (13)
ET	MM	7 Months	67/F	1,220	Not performed	IgG kappa	NR	Selroos, 1984 (13)
ET	MM	5 Years	66/M	940	1st: Mega-K, 1% PC 2nd: 50% PC	IgG kappa	NR	Prosper, 1992 (14)
ET	MM	7 Years	50/F	1,152	1st: Mega-K 2nd: PC	IgG kappa	NR	Kelsey, 1995 (15)
ET	MM	10 Years	83/F	1,500	1st: Mega-K 2nd: 50% PC	IgA kappa	NR	Arlen,1995 (16)
ET	MM	5 Years	68/F	697	1st: Mega-K 2nd: 24% PC	IgA lambda	NR	Cobo, 1995 (17)
ET	MM	5 Years	85/M	927	1st: Mega-K 2nd: 60% PC	IgG lambda	NR	Majhail, 2003 (18)
ET	MM	3 Years	54/M	900	1st: Mega-K 2nd: 40% PC	IgG kappa	NR	Majhail, 2003 (18)
ET	MM	3 Years	48/F	2,000	100% PC	IgA kappa	NR	Eskazan, 2011 (19)
ET	MM	6 Years	66/M	2,770	1st: MPN 2nd: 20% PC	IgG kappa	JAK2+	Youssef, 2013 (20)
ET	MM	3.5 Years	52/M	1,130	1st: Mega-K 2nd: 80% PC	Non-Secretory	JAK2-	Lekovic, 2013 (21)
ET	MM	4 Years	71/M	840	1st: Mega-K 2nd: 60% PC	IgG kappa	JAK2+	Badelita, 2014 (22)
ET	MM	2 Years	68/M	1,000	1st: Mega-K 2nd: 20% PC	IgA lambda	JAK2+	Terzi, 2015 (23)
ET	MM	5 Years	69/M	688	1st: Mega K 2nd: 30% PC	IgG lambda	JAK2-	Loscocco, 2021 (24)

ET, essential thrombocythemia; MM, multiple myeloma; MP, monoclonal protein; NR, not reported; Mega-K, megakaryocyte proliferation; PC, plasma cell; FLC, free light chain.

Reports prior to 2011 did not describe JAK2 status or CALR mutations. Additionally, none of the previous reports of synchronous MM and ET had simultaneous treatment for both malignancies, as was required in our case. Although the exact mechanism of such SDHMs is not clearly understood, there are several possible reasons. Firstly, a common trigger at the stem cell level may lead to their differentiation into myeloid (ET) and lymphoid (MM) cells (12). Secondly, the two malignancies may arise from separate malignant clones at different differentiation levels (18). Alternatively, therapy for one malignancy may cause or trigger development of the second malignancy (25); however, this is unlikely to be the case in SDHMs, as in our patient. Additionally, it has been suggested that interleukin-6 may cause reactive thrombocytosis via stimulation of thrombopoietin and may precipitate synchronous development of ET (26). It is also possible, however, that the occurrence of the two malignancies was purely coincidental. In our case, the patient had a history of coronary artery disease with myocardial infarction 4 years before diagnosis. However, given the history of hypertension and hyperlipidemia as well as the timing, previous cardiovascular events are less likely to be related to ET. MM is a slowly progressing malignancy, with an average time gap of 163 days from symptom onset to diagnosis (27); thus, it may have been present subclinically for some time in our patient before its diagnosis meaning that these malignancies may not necessarily have been synchronous. Due to the concomitant development of ET and MM in our patient, it is likely that BM infiltration by PC suppressed excessive megakaryopoiesis and thus masked ET. With restoration of hematopoiesis by antimyeloma therapy, the patient developed thrombocytosis. In such cases when clinical or biochemical parameters cannot be explained by the primary malignancy alone, it is important to consider DHMs. MCV could be one of the clues pointing to DHMs: in our patient, macrocytosis at diagnosis was most likely related to MM since other causes (hypothyroidism, liver disease, reticulocytosis, B12 deficiency, etc.) had been excluded. With treatment of MM, MCV normalized and reappeared with initiation of HU.

In managing such patients, one needs to treat the malignancy that has a more aggressive, life-threatening course or the potential to transform into acute leukemia. In our case, we first treated for MM. Unexpected extreme thrombocytosis prompted addition of cytoreductive therapy for ET. Additionally, therapy for DHMs needs to balance disease control, the patient’s condition, and BM capacity. We did not observe any effect of treatment for MPV, Ld, or daratumumab on ET. With the fourth-line PCd, the patient had normalization of platelets but not to the level of thrombocytosis; possibly due to “tired bone marrow” with decreased capacity to produce excess platelets or due to antiplatelet effects of PCd. Similarly, therapy with HU for ET did not have any effect on the course of MM. Duration of response with all therapies in our patient was comparable to that of previously reported patients with MM alone. Despite our patient’s age, comorbidities, and combination of treatments, she tolerated multiple lines of treatment well for both malignancies and achieved CR.

## 4 Conclusion

Synchronous MM and ET are rare. BM suppression by MM can mask thrombocytosis at diagnosis. Restoration of hematopoiesis due to antimyeloma therapy may uncover ET. Therapy of MM does not affect ET, except probably use of PCd. Frequent monitoring of both malignancies is important, as drops in blood counts could be a result of either cytoreductive therapy or MM relapse. Presence of concomitant ET did not decrease efficacy of sequential antimyeloma therapies in our elderly and frail patient.

## Data availability statement

The original contributions presented in the study are included in the article/supplementary material. Further inquiries can be directed to the corresponding author.

## Ethics statement

Ethical review and approval was not required for the study on human participants in accordance with the local legislation and institutional requirements. The patients/participants provided their written informed consent to participate in this study. Written informed Consent for Publication was obtained from the patient’s daughter (next-of-kin) for publication of the details of their medical case and any accompanying images.

## Author contributions

NK, RP, and RK wrote and reviewed the manuscript. All authors agreed to act as guarantors of the work. All authors contributed to the article and approved the submitted version.
